# The use of *Lespedeza cuneata* for natural control of gastrointestinal nematodes in Merino sheep

**DOI:** 10.4102/ojvr.v84i1.1259

**Published:** 2017-04-11

**Authors:** Erika A. van Zyl, Francien S. Botha, Kobus J.N. Eloff, Phumzile P. Msuntsha, Peter A. Oosthuizen, Cathy Stevens

**Affiliations:** 1 Dundee Research Station, KZN Department of Agriculture and Rural Development, South Africa; 2 Department of Paraclinical Sciences, University of Pretoria, South Africa

## Abstract

*Lespedeza cuneata* (poorman’s lucerne; sericea lespedeza), a tannin-rich perennial legume, was offered as hay to dry Merino ewes in a confined feeding experiment to evaluate the effect on the level of gastrointestinal parasite infection in sheep. *Medicago sativa* (a low tannin containing perennial legume) was used as the control treatment. Parameters faecal egg count (FEC), FAMACHA^©^ scores and rectal temperatures were used. FECs were substantially lower (*p* = 0.05) in the *Lespedeza* group after 35 days, together with a trend of higher rectal temperatures, compared with the *Medicago* group. Although non-significant (*p* > 0.05), the higher rectal temperatures suggested a lower level of anaemia in the sheep on the *Lespedeza* ration and, therefore, a lower parasite-worm burden. However, FAMACHA^©^ scores showed no significant (*p* > 0.05) differences between treatments despite the differences in FEC that were recorded, indicating that host homeostasis was possibly mediated by improved nutrition as a result of the high protein content of both experimental diets.

## Introduction

The existence of livestock is closely bound to that of parasites (Villalba et al. 2014). Increased concentrations of livestock and monoculture foraging have enhanced gastrointestinal parasite populations to such a level that livestock production has, to a large extent, become dependent on anthelmintic chemotherapy. The extensive use of these synthetic anthelmintics to control gastrointestinal nematode (GIN) infections, driven by frequency of treatment and underdosing of animals, has resulted in the development of widespread nematode resistance to chemical anthelmintics (Besier & Love 2003; Greef, Karrison & Schlink 2014; Morgan et al. 2013; Preston et al. 2014; Taylor, Hunt & Goodyear 2002). The most important strongylid nematodes in the tropics and subtropics that cause severe economic losses in sheep farming is *Haemonchus contortus* (Adamu 2012; Adamu, Naidoo & Eloff 2013; Vatta & Lindberg 2006), which showed resistance in the benzimidazole group for the first time in South Africa in 1975 (Berger 1975). Since then, the levels of resistance in South Africa are among the highest in the world (Van Wyk et al. 1999; Vatta & Lindberg 2006).

The increased drug resistance of parasites to modern anthelmintics, together with the increased public awareness of drug residues in animal products, motivated the search for alternative endoparasite control strategies in small stock. Research has identified promising alternative anthelmintics in several well-known pasture species, the so-called bioactive forages, such as chicory (Athanasiadou et al. 2007; Foster, Cassida & Turner 2011; Heckendorn 2007), sainfoin (Heckendorn 2007; Hoste et al. 2014), sulla (Niezen et al. 1995, 1998) and sericea lespedeza (Lange et al. 2006; Moore et al. 2008; Shaik et al. 2006). These pastures have a tremendous advantage over many other plants with anthelmintic activities, because they are already established as planted pastures and available on farms for direct consumption by animals as grazing or as preserved feed as hay.

Of specific importance are the tanniferous forages, where indications are that condensed tannins (CTs) play a cardinal role in the anthelmintic activities. Generally, tannins will defend the plant against utilisation by lowering the palatability and negatively influencing digestion. The slowdown in digestion caused by tannins can, in turn, also lower intake because of gut fill (Barry & McNabb 1999; Hovarth 1981; Rahmann & Seip 2007; Reed et al. 1982; Shimada 2006).

These negative effects are generally associated with forages that contain high CT concentrations of above 55 g CT kg^−1^ dry matter (DM) (Min & Hart 2003). Diets with moderate concentrations of CT (20 g CT kg^−1^ – 40 g CT kg^−1^ DM) may lead to improved protein nutrition when tannins complex with plant proteins, resulting in increased protein assimilation by safeguarding some proteins past the rumen to the rest of the intestines, instead of being lost through secretion (Hoste et al. 2006; Min & Hart 2003).

CTs may thus have an indirect effect on GIN, by replenishing the extensive endogenous protein losses encountered in the abomasum and small intestine of the host during GIN parasitism, thus enhancing immunity or improving host homeostasis (Bown, Poppi & Sykes 1991; Hoste et al. 2006; Min & Hart 2003; Niezen et al. 2002).

The mechanism of activity of direct effects of tannins on parasites is less clear than the indirect effects. Electron microscope investigations showed cuticular changes after contact with tannins, which may inhibit or delay the exsheathment of the L_3_ stage after indigestion by the host (Hoste et al. 2006). A hypothesis was put forward that CT bonding takes place with the proline- and hydroxyproline-rich cuticle that covers the body and lines certain parts of the digestive tract and reproductive system of the nematode (Hoste, Torres-Acosta & Aguilar-Caballero 2007).

Several workers reported repressed female nematode reproductive activity as another direct effect of CT on GIN, which explained the decrease in faecal egg count (FEC) measured in several trials where feeding experiments were conducted with tannin-rich forages (Ahmed, Laing & Nsahlai 2010; Heckendorn 2007; Lange et al. 2006; Min & Hart 2003; Min et al. 2005; Niezen et al. 1998, 2002; Shaik et al. 2004, 2006). However, research done on goats by Shaik et al. (2006) showed that a CT diet had highly significant (*p* < 0.001) effects on reducing the numbers of adult nematodes in both the abomasum and small intestines, and that female nematodes were more affected.

*Lespedeza cuneata*, also known as sericea lespedeza or poorman’s lucerne, is a CT-containing pasture that has anthelmintic properties (Ahmed et al. 2010; Lange et al. 2006; Min & Hart 2003; Min et al. 2005; Shaik et al. 2004, 2006; Terrill et al. 1989). Significant outcomes were measured regarding a reduced FEC and increased packed cell volume, but insufficient on-farm implementation strategies were recommended (Burke et al. 2011; Gujja et al. 2013; Kommuru et al. 2014; Shaik et al. 2004, 2006; Terrill et al. 2007). Many of the studies were done on goats.

An advantage of *L. cuneata*, above several other plants with confirmed anthelmintic properties, is that it is already a commercially established pasture with seed available, is relatively drought resistant and is adaptable to low fertility soils (Dannhauser 2002). Being a legume, it has nitrogen-fixing abilities, which make it a low input pasture. The main objective of the current investigation was to determine the effects of feeding *L. cuneata* leaf hay on the level of an established gastrointestinal parasite infection in Merino sheep as measured by FEC. The second objective was to investigate whether changes in the level of GIN infection, if any, could be detected by changes in rectal temperature and FAMACHA^©^ scoring as an indication of anaemia. Anaemia, as a result of a loss of haemoglobin due to parasitism, is associated with low body temperature caused by a restricted oxygen and iron supply to cells in the body, which in turn, interferes with the ability of the body to regulate its temperature.

*Medicago sativa* (commonly known as lucerne or alfalfa), a perennial legume pasture, was used as the control forage. It has a low CT content and lacks anthelmintic properties and is often used as contrasting forage to high-tannin forages in parasite-forage studies (Valderrábano, Calvete & Uriarte 2010).

## Materials and methods

### Experimental animals and design

A confinement feeding trial was conducted with dry, non-pregnant Merino ewes at the Dundee Research Station, KwaZulu-Natal (KZN) Department of Agriculture and Rural Development, South Africa. The sheep were housed in covered barns with open sides and concrete flooring in pens, each covering an area of 24 m^2^. The pens had similar temperature and sunlight conditions.

The ewes were grazed on rain-fed Nile grass (*Acroceras macrum*) pasture prior to the commencement of the feeding trial to acquire a low-level GIN infection. Weekly FEC, expressed as eggs per gram (EPG) of individual sheep, verified their GIN infection status. Sufficient levels of infection (mean ± 3000 EPG) were reached after 35 days, and no trickle infection with parasite larvae was needed to boost the level of infection.

Animals (*n* = 14) were then ranked according to their FEC status and assigned to one of two treatment groups. Each treatment was replicated twice. The ewes in each treatment were then allocated to two pens with either three or four sheep in each pen. The pens were, for the duration of the trial, carefully cleaned each day to prevent GIN reinfection.

All husbandry practices and experimental procedures were approved by the applicable authorities. The animals were on a salvaged deworming protocol, that is, deworming with an effective chemical dewormer. This was done using an FEC reduction test prior to the trial. Treatment would take place if the FEC in an individual sheep exceeded a level of 6000 EPG over two consecutive weeks. No animals required salvage deworming throughout the study.

### Experimental diets

The experimental diets were:

- *Lespedeza cuneata* hay (L group).
- *Medicago sativa* hay (M group).

The *L. cuneata* material was obtained from hay made from a pure *L. cuneata* pasture at Dundee Research Station. The *M. sativa* hay for the trial was brought in from a local commercial producer.

Sheep are selective grazers, and it was observed that sheep selectively graze or ‘browse’ *L. cuneata*, selecting the leaves and avoiding the stems. Therefore, in the experiment, only the leaf part of the hay was used. Stems were removed semi-mechanically.

Random samples from the forages were taken for feed analysis. Samples were milled through a 2-mm sieve and sent for full feed chemical analysis according to methods described by De Figueiredo and Thurtell (1998), which are based on the Van Soest’s (1965) methods. The analyses were done at the Cedara Feed Laboratory of the KZN Department of Agriculture and Rural Development. The results of these analyses were used to nutritionally balance the rations. The *Lespedeza* ration had to be supplemented and consisted of 80% *L. cuneata* leaves, 12% high protein concentrates and 8% molasses meal (molasses meal from Molatek: protein = 40 g kg^−1^; metabolisable energy = 105 MJ kg^−1^). The ration of the M group consisted only of *M. sativa* hay.

For the CT analysis, a random sample was collected from the harvested *L. cuneata* forage material and air-dried in the shade. Samples were divided by hand into stem and leaf samples for reasons discussed above. These samples were analysed for tannin content by the Cedara Feed Laboratory, according to a method described by Reed et al. (1982) and Waterman and Mole (1994). The CTs were extracted from the samples with aqueous acetone (70% acetone). Afterwards, the butanol–HCl and ferric reagents were added, followed by measuring the absorbance at 550 nm with a spectrophotometer. CT levels were expressed as g CT kg^−1^ DM.

The experimental diets were fed daily *ad libitum* on a pen basis, for 35 days. The daily rations allow for 10% remaining feed. Fresh drinking water was provided daily *ad libitum.*

### Sampling procedures and analysis

The live body weights of sheep were taken at commencement of the trial and weekly thereafter. Weighing was done without fasting the animals, but always in the early morning before the daily ration was offered.

Fresh dung samples were collected directly from the rectum of individual sheep for FEC analysis. Samples were collected prior to the start of the experiment to determine the level of GIN infection and then at the commencement of the trial to aid in the randomisation of the animals. Thereafter, collections were made weekly until Day 35, when the experiment was terminated.

Faecal samples (10 g animal^−1^ – 15 g animal^−1^) were analysed for FEC using the modified McMaster and Visser slide technique (Hansen & Perry 1994). Three grams of the collected faeces were diluted in 30 mL of saturated sugar solution. After mixing, a sample was taken with a pipette and dropped into the McMaster slide chambers. Using a microscope, ova were counted on both sides of the chamber and multiplied by 50 to estimate the total number of eggs in a sample, as EPG of faeces.

Rectal temperature was taken with a handheld thermometer (Digiflash; Pfizer). Rectal temperatures were taken early in the morning, and care was taken that the animals were not exposed to abnormal activities before readings were taken.

FAMACHA^©^ scoring is an indirect measurement of the level of anaemia, as observed by the colour of the mucous membrane of the eye, which is assessed using a colour guide chart. A scoring of between 1 and 5 is used, where 1 is healthy, pink-red colour and 5 is white mucous membranes, indicating severe anaemia as a result of parasitism (Van Wyk & Bath 2002).

Animal weights, FAMACHA^©^ scores and rectal temperatures were taken simultaneously with dung sample collection.

### Statistical analysis

The data on live mass of sheep, FEC, FAMACHA^©^ and rectal temperatures were analysed using the statistical program Genstat (Payne et al. 2011). Repeated measures analysis of variance was used for the FEC, live mass and rectal temperature, which were measured over time. Log transformations were done on the FEC data to stabilise variance. Fisher’s protected test of least significant differences (LSD) was conducted at a 5% significance level. Multiple regression analysis was used to test for relationships.

## Results

### 

#### Chemical composition of feed and intake

The results of the analyses of chemical composition and the CT content of the *M. sativa* hay, which made up the ration of the M group, were 16.55% crude protein (CP), 44.52% neutral detergent fibre (NDF) and 36.12% acid detergent fibre (ADF), with the CT content of < 1.5 g kg^−1^ DM. The results for the L group were 17.17% CP, 41.91% NDF and 30.45% ADF, with the CT content of 80 g kg^−1^ DM. The results are presented in Table 1. Voluntary intake over the trial period was similar for both groups.

**TABLE 1:** Crude protein, fibre (neutral detergent fibre and acid detergent fibre) and condensed tannin levels of the rations fed to sheep.

Parameter	L group ration	M group ration
CP (%)	17.17	16.55
NDF (%)	41.91	44.52
ADF (%)	30.45	36.12
CT (g kg^−1^ DM)	80.00	< 1.50

CP, crude protein; NDF, neutral detergent fibre; ADF, acid detergent fibre; CT, condensed tannins.

#### Live weight

The average live body weight of the sheep at commencement of the trial was 54.90 kg ± 5.78 kg. Ewes in the L group had an average daily gain (ADG) of 0.08 g sheep^−1^ day^−1^, and ewes in the M group had an ADG of 0.09 g sheep^−1^ day^−1^. Treatments did not differ significantly (*p* > 0.05), but the interaction between treatment and time, indicating a live weight increase over time, was significant (*p* < 0.05).

#### Faecal egg count

**FIGURE 1:**
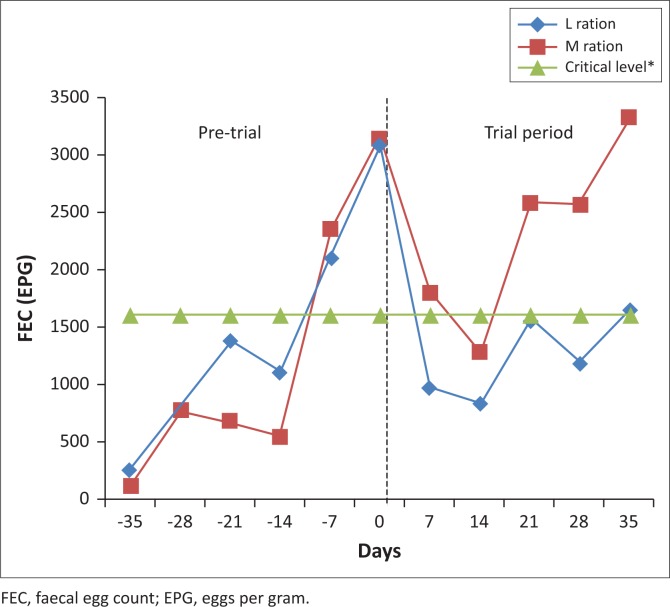
The effect of feeding a *Lespedeza cuneata* or *Medicago sativa* hay ration on the faecal egg count of parasitised sheep in the trial with indication of the critical faecal egg count level according to Colditz 2008.

The mean FEC, 3086 EPG for the L group and 3143 EPG for the M group at the start of the experiment, decreased in both groups following commencement of feeding up to Day 14 (829 EPG for the L group and 1286 EPG for the M group). The FEC then gradually increased in both groups from Day 14 to Day 21, with the levels of 1557 EPG for the L group and 2586 EPG for the M group. A slight decrease on Day 28 and then an increase on Day 35 for the L group were recorded with the final mean FEC of 1643 EPG for the L group and 3329 EPG for the M group. FEC levels in the M group followed the same trend compared to the L group, but at higher levels of FEC (Figure 1).

FEC levels in the two treatments were not significantly (*p* > 0.05) different up to Day 28 but were significantly different by Day 35 (*p* = 0.05), with high variability in the data. Log_e_ transformations were used to stabilise the variance in FEC (Table 2). FEC changes over time were highly significant in both treatment groups (*p* < 0.001).

**TABLE 2:** The faecal egg count results (Log_e_ transformations) of sheep on different days on a *Lespedeza cuneata* or *Medicago sativa* hay ration.

Days	FEC (Log_e_ transformations) for sheep fed different rations		SEM	LSD of means (5% level)	*p*
	*Lespedeza*	*Medicago*			
Day 7	6596	7130	0.37	1.15	> 0.05
Day 14	6316	7064	0.31	0.97	> 0.05
Day 21	6647	7664	0.45	1.39	> 0.05
Day 28	6744	7608	0.36	1.10	> 0.05
Day 35	7139	7981	0.28	0.86	= 0.05

FEC, faecal egg count; LSD, least significant difference.

#### Rectal temperature

**FIGURE 2:**
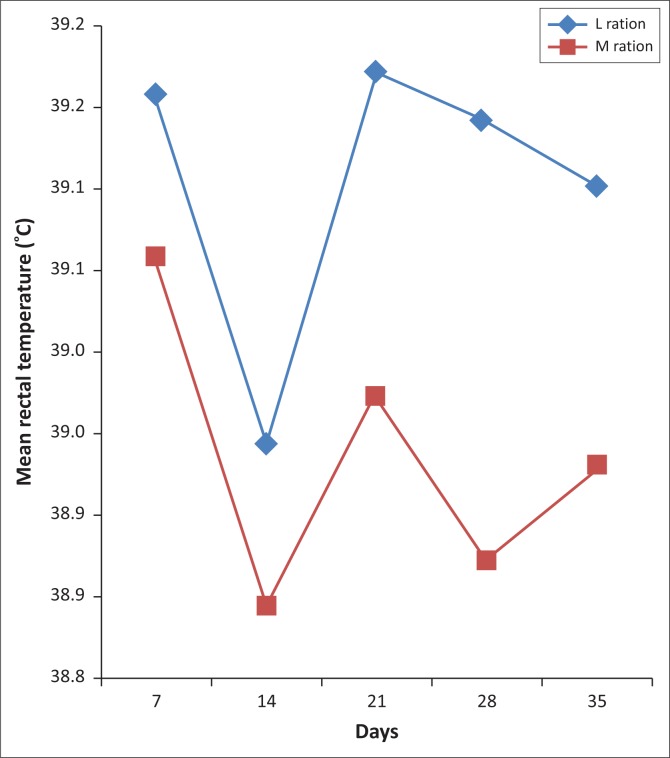
The effect of feeding *Lespedeza cuneata* or *Medicago sativa* hay on the mean rectal temperature (°C) of parasitised sheep.

The normal rectal temperature of sheep varies between 38.3 °C and 39.9 °C. The rectal temperatures of the sheep on trial were monitored to investigate whether differences could be detected between the rectal temperatures of sheep in the treatment groups with different parasite loads (Figure 2). For the duration of the experiment, the measured rectal temperatures were within the normal range. As temperatures were taken during the cool early morning, air temperatures were not taken into consideration.

The rectal temperature in the M group was consistently lower for the duration of the experiment compared with the L group, although the difference was not significant (*p* > 0.05). A decrease in rectal temperature was observed on Day 14 of the experiment, which coincided with a lower level in the FEC. The explanation of this observation is unclear.

The differences in mean rectal temperature between the treatments were not significant at the 5% level (*p* = 0.05), but there was a trend for lower temperatures on Day 21 (*p* < 0.07) and on Day 28 (*p* < 0.08; Table 3). No significant relationship existed between rectal temperature and FEC over the trial period.

**TABLE 3:** The rectal temperatures (°C) of sheep on a *Lespedeza cuneata* or *Medicago sativa* hay ration.

Time	Rectal temperature (°C) for sheep fed different rations		*p*	LSD of means (5% level)	SEM
	*Lespedeza*	*Medicago*			
Day 7	39 157	39 057	0.59	0.397	0.129
Day 14	38 943	38 843	0.55	0.352	0.114
Day 21	39 171	38 971	0.07	0.220	0.071
Day 28	39 143	38 871	0.08	0.081	0.310
Day 35	39 100	38 929	0.12	0.222	0.072

LSD, least significant difference.

**FIGURE 3:**
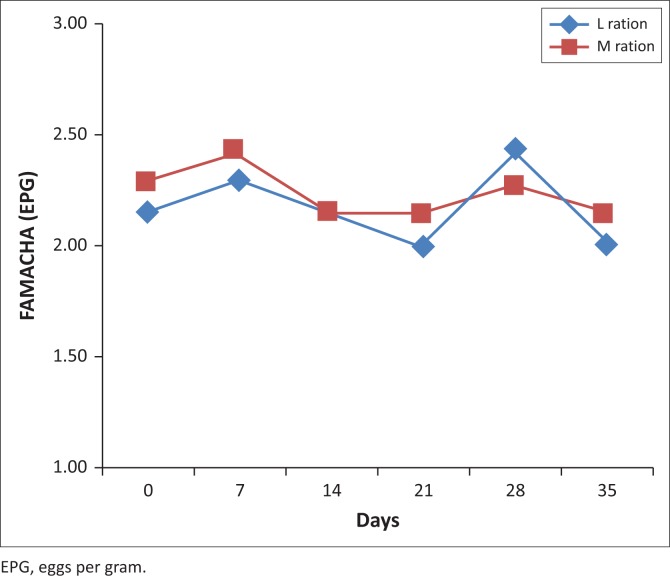
The effect of feeding a *Lespedeza cuneata* or *Medicago sativa* hay ration on the mean FAMACHA^©^ scores of parasitised sheep.

No significant (*p* > 0.05) differences were detected in the FAMACHA^©^ scores. The scores also did not pick up the changes in infection as shown in the FEC results. The low FAMACHA^©^ scores indicated that sheep were only slightly anaemic (Figure 3).

## Discussion

Feeding of a *Lespedeza* leaf hay ration to parasitised sheep appears to be an effective means of suppressing GIN infection in mature Merino sheep. The FEC levels between the treatments in the current investigation decreased by 47% in the L group compared with an increase of 6% in the M group compared with the levels at the commencement of the trial.

The reduction in FEC observed in the L group confirms results from previous studies where *L. cuneata*, fed in dried form (hay, leaf meal and pellets), resulted in reduced FEC. Lange et al. (2006) investigated the effect of *Lespedeza* hay on the worm burdens in lambs. The worm burden was reduced by 67% and the establishment of incoming larvae was reduced by 26%. Shaik et al. (2006) tested *L. cuneata* hay as a natural deworming agent against GINs in goats and found that FECs were significantly reduced, compared with goats fed *Cynodon dactylon* hay. Kommuru et al. (2014) reported a decreased FEC within 1 week of feeding a 90% *L. cuneata* ration to GIN-infected kids, but Terrill et al. (2007) reported an initial delayed reaction on the effect on egg production when feeding *L. cuneata* hay to goats in a titration study.

The L ration in the current study consisted of 80% *Lespedeza* leaves. Burke et al. (2011) did a *L. cuneata* dose titration study on lambs. *Lespedeza cuneata* meal was fed to *H. contortus*-infected lambs in diets containing 0%, 25%, 50% and 75% *Lespedeza* and concluded that FEC was not influenced by the *L. cuneata* fraction in the diet.

Min and Hart (2003) compared the results of several studies regarding the effects of CT levels on FEC reduction. When CT levels increased above 55 g kg^−1^ DM and decreased below 45 g kg^−1^ DM, the FEC responses became variable, but between these levels (45 g – 55 g of CT kg^−1^ DM), fairly constant FEC reductions of ± 50%, relative to non-CT containing forages, were reported. More specifically, *L. cuneata*, with CT content of 50 g kg^−1^ DM, had shown to reduce FEC by 60%.

The *Lespedeza* leaves in the current study had a CT content of 80 g kg^−1^ DM, which exceeds the critical level of 55 g kg^−1^ DM mentioned above, after which FEC response seemed to become variable. However, this level is well within the range of 46 g kg^−1^–152 g kg^−1^ DM mentioned by Coffey et al. (2007) as expected levels of CT in *L. cuneata*. In comparison, these authors reported expected CT levels of 0.5 g kg^−1^ DM for *M. sativa*, 48 g kg^−1^ DM for Birdsfoot trefoil, 77 g kg^−1^ DM for Big trefoil and 29 g kg^−1^ DM for sainfoin.

The results of the current study also correspond fairly well with similar experiments conducted by Niezen et al. (1995, 1998), where the FEC results of parasitised lambs on *Hedysarum coronarium* (sulla), a high CT-containing forage (CT of 110 g kg^−1^ DM), and on *M. sativa*, a low CT-containing forage (CT of 1.5 g kg^−1^ DM), were compared. The effect of grazing was significantly different (*p* < 0.01) for FEC; differences of 1320 EPG for *H. coronarium* were recorded, compared with an FEC of 2200 EPG for *M. sativa.* A total worm burden of 10 553 for *H. coronarium* was measured, compared with 18 676 for *M. sativa* (Min & Hart 2003).

Several authors reported that the tannins in tannin-rich forages become more polarised when field-dried, resulting in a lower number of free hydroxyls available for binding proteins, which implies that, in hay form, safe intake levels could be higher when compared with green grazing (Barry & McNabb 1999; Hovarth 1981; Reed, Soller & Woodward 2003; Terrill et al. 1989). These ‘unbound’ CTs are most likely the active agent giving the plant its anthelmintic properties. The molecular structure of CT in the plant is more critical to its anthelmintic properties than the concentration of different CT types (Shaik et al. 2006).

GIN, voracious bloodsucking endoparasites living in the digestive tract of their host, creates extensive damage to the gastrointestinal mucosa. This results in increased plasma leakage and losses of endogenous protein, as well as interference with the retention of nitrogen, vitamins and minerals (Kimambo et al. 1988; Villalba et al. 2014). Colditz (2008) reported that a blood loss of 10 mL day^−1^ is typical of what parasitised mature sheep with an FEC of 1600 EPG experienced. Blood loss in excess of this norm will result in anaemia. The results in the current study showed that the FEC in the L group were consistently below this norm from Day 7, which was not the case in the M group. These results suggested that the sheep in the M group would have required anthelmintic treatment at the termination of the trial, compared with sheep in the L group where treatment was not required.

The trend of higher rectal temperatures in the L group, corresponding with the trend in FEC in the two treatments, suggests a possible lower level of anaemia in the L group compared with the M group. However, Specht (1982), after an investigation into the effect of GIN infections on host-body temperature, concluded that body temperature results did not show as close a relationship to parasite infection as expected.

FAMACHA^©^ scoring, a well-known indicator of the level of anaemia in small stock, did not detect any differences between the two treatment groups over the trial period. This lack of anaemia, despite the FEC results, was possibly mediated by improved nutrition as a result of the high protein content of both experimental diets, which replenished the endogenous protein losses encountered during GIN parasitism. This is supported by the work by Van Houtert and Sykes (1996) who showed that host resilience or the ability of the animal to withstand the effects of infection can be markedly enhanced by increasing the metabolisable protein supply, which in the case of the current study was supplied by both forages used in the trial.

The reduced FEC results in the L group could be a direct or indirect effect of the CT on the GIN, either by reduced female parasite egg shedding or reduced numbers of parasites. Both Shaik et al. (2006) and Lange et al. (2006) attributed reduced FEC to lower parasite numbers and a reduction in worm fecundity.

The lasting anthelmintic effects on GIN after cessation of the CT-containing feed appear to be variable. Heckendorn (2007) reported a sustainable reduction in FEC, but other studies showed that reduction in EPG disappeared when CT-containing feeding was stopped (Coffey et al. 2007; Lange et al. 2006; Min et al. 2004), indicating that worms were inhibited and not killed. Athanasiadou and Kyriazakis (2000) suggested that CT only temporarily reduced female worm fecundity.

Goats and sheep differ in their level of tolerance to the effects of CT. The more protein-rich saliva excreted by goats while eating is thought to be the first defence against tannins, compared with sheep. Goats are generally more tolerant of CT than sheep, hence their browsing habits (Hoste et al. 2007; Min & Hart 2003; Mueller-Harvey 2006). Therefore, care must be taken to not equate the anthelmintic-related results from goat studies directly to sheep.

## Conclusion

The study proved that by feeding *L. cuneata* hay to GIN-infected sheep, the level of infection, as measured by FEC, decreased substantially. Rectal temperatures and FAMACHA^©^ scoring as an indication of anaemia level of GIN infection could not detect the changes in GIN infection. This was most possibly due to the high protein content of the experimental diets, which curbs endogenous protein losses as a result of GIN parasitism. The proven efficacy of *L. cuneata* hay as an anthelmintic suppressor holds exciting possibilities for sheep farmers. *Lespedeza cuneata*, as an already established planted pasture with agronomic advantages, such as nitrogen-fixing abilities, relative drought resistance and adaptability to low fertility soils, together with anthelmintic properties, can therefore, be advantageously and flexibly incorporated in the fodder flow programmes on sheep farms. In addition to providing a high protein content forage, *L. cuneata* can be strategically offered to animals when a GIN challenge is expected, such as when weather becomes favourable for the development of GIN infections. Further, with less GIN egg shedding, pasture contamination will decrease future infections.
